# Emotional Contagion in the Workplace: A Moderated Mediation Model of Psychological Well-Being, Job Performance, and Turnover Intention in Hotels

**DOI:** 10.3390/ejihpe16040050

**Published:** 2026-03-31

**Authors:** Alaa M. S. Azazz, Ibrahim A. Elshaer, Hemdan El-Shamy, Sameh Fayyad, Osman Elsawy, Abuelkassem A. A. Mohammad

**Affiliations:** 1 Department of Social Studies, Arts College, King Faisal University, Al-Ahsa 31982, Saudi Arabia; 2 Department of Management, College of Business Administration, King Faisal University, Al-Ahsa 31982, Saudi Arabia; 3 Department of Psychology, Statistics and Educational Evaluation, Faculty of Education, King Faisal University, Al-Ahsa 31982, Saudi Arabia; helshamy@kfu.edu.sa; 4 Hotel Management Department, Faculty of Tourism and Hotels, Suez Canal University, Ismailia 41522, Egypt; sameh.fayyad@tourism.suez.edu.eg; 5 Department of Human Resources Management, College of Business, King Khalid University, Abha 61471, Saudi Arabia; osman.alsawy@tourism.suez.edu.eg; 6 Faculty of Tourism and Hotels, Minia University, Minia 61519, Egypt; kassem.mohammad@mu.edu.eg; 7 Faculty of Tourism and Hospitality, King Salman International University, Sharm EL Sheikh 16949, Egypt

**Keywords:** emotional contagion, workplace, employee performance, leader–member exchange, psychological well-being, employee’s turnover intention

## Abstract

The hotel industry is widely induced by emotional transactions between frontline employees and their guests leading to unintentional transfer of emotions, a phenomenon known as emotional contagion (EC). EC can result in positive or negative outcomes in the workplace influencing employees’ well-being and performance. This research paper explored direct effects of emotional contagion (EC) on (H1) employees’ well-being (PW), (H2) job performance (JP), and (H3) turnover intention (IL). Based on the affective events theory (AET) and the social exchange theory (SET), employee’s psychological well-being was employed as a mediating factor (H6-H7) and leader–member exchange (LMX) as a moderator variable that might alleviate the adverse consequences of EC (H8). Cross-sectional survey data were collected online from 792 frontline employees. The proposed model was evaluated with partial least squares structural equation modeling (PLS-SEM). The findings revealed that EC can significantly weaken PW, which accordingly decreases JP and increases IL. Nonetheless, strong levels of LMX can alleviate these harmful influences, emphasizing the main significant role of LMX in regulating emotional dynamics in the service workplace. This study expands our understanding of how emotional mechanisms and LMX practices can adjust employee resilience, retention, and performance in the context of high-emotion service.

## 1. Introduction

Human resources management (HRM) is among the key assets in the service industry, as it incorporates both emotional and intellectual factors. With the ongoing evolution of the service sector and the growing significance of staff–customer interactions, staff emotions have become a key issue in delivering high levels of customer service (Aboutaleb et al., 2025). In this setting, frontline hotel employees are expected to manage their own feelings and express only positive behaviors, such as friendliness and warmth (Li et al., 2018). At the same time, frontline service employees witness different emotions during interactions with colleagues, superiors, and consumers, which, in turn, can reshape their effective behavior and states (Groth et al., 2019). These dynamics echo the process of emotional contagion, a phenomenon depicted by (Hatfield et al., 1994) in which people “catch” others’ emotions. People with enhanced affective sensitivity are particularly susceptible, as they are more reactive to emotions conveyed, often unintentionally by others (Bellamkonda, 2025). That is, hotel employees at services encounters are prone to be affected by the emotions experienced by guests and co-workers due to emotional contagion (Wu & Wu, 2019).

In the post-pandemic era of COVID-19, hotel frontline employees have become more exposed to heighted emotional encounters as a result of health-related issues and safety concerns alongside increased guest expectations (Baum et al., 2020; Karatepe et al., 2021). As such, emotional contagion has become more prevalent as service employees tend to unintentionally absorb and mirror the overwhelming emotions they catch from guests and their colleagues. Previous studies in hospitality and organizational psychology (Baum et al., 2020; Karatepe et al., 2021; Wu et al., 2024) indicated that these conditions led to emotional strain and job burnout among frontline employees. Accordingly, emotional contagion represents an interesting phenomenon that depicts the mechanism of transforming negative emotions and pressure into diminished employees’ well-being.

The affective events theory (AET) further describes that workplace outcomes can elicit both negative and positive emotional responses, which, in turn, impact employees’ psychological states, behavior, and attitudes (Chaudhry & Espinosa, 2024). Notably, staff regularly internalize adverse emotions, whether directly observed or experienced by others, leading to detrimental consequences such as burnout, emotional exhaustion, reduced commitment, and reduced engagement (Shamshad et al., 2025). Such emotional strain can weaken psychological well-being (Verzeletti et al., 2016), impair employees’ job performance (Reizer et al., 2019), and improve turnover intentions (Obeng et al., 2025).

Hotels should handle emotional contagion between frontline employees to avoid its negative consequences. Guided by JD–R theory, emotional demands arising from contagion can harm behavior but are buffered by resources, such as strong leader–member exchange (LMX). A further resource-based explanation is offered by integrating COR theory, which demonstrates how psychological well-being functions as a crucial mediator converting emotional demands into behavioral results (Aslam et al., 2022). The concept of resource caravans is also highlighted by COR theory, which contends that beneficial connections like excellent leader–member exchange (LMX) can allow workers to gather and utilize several resources at once (Aboutaleb et al., 2025; Elshaer et al., 2025). This mechanism, which emphasizes how relational resources may mitigate the detrimental effects of emotional contagion, supports the theoretical justification for the moderating role of LMX (Chang et al., 2026). Similarly, COR theory highlights employees’ drive to acquire and protect resources, with supportive leader–employee ties serving as a key buffer against contagion’s effects (S. E. Hobfoll et al., 2018). According to COR theory, workers are driven to perform tasks that advance their values, such as well-being, and therefore become more inclined to make decisions that optimize their well-being. Research indicates a positive correlation between employee well-being and organizational support provided by superiors and direct managers. Effective working connections between supervisors and coworkers can provide adequate assistance that enhances employee well-being (Nisar & Maroulis, 2017). Over time, such a profound relationship creates a mutual reciprocity that benefits both the employees involved and the firm (Cropanzano et al., 2017). By integrating COR theory, the concept of resource caravans is also highlighted. Resource caravans contends that beneficial connections like excellent leader–member exchange (LMX) can allow workers to gather and utilize several resources at once. This mechanism, which emphasizes how relational resources may mitigate the detrimental effects of emotional contagion, supports the theoretical justification for the moderating role of LMX.

Recently, there has been a growing number of studies on emotional contagion in the tourism and hospitality industry domain (Aboutaleb et al., 2025; Elshaer et al., 2025; Herrando et al., 2022; Hofmann et al., 2024; Hsiao et al., 2022; Schoner-Schatz et al., 2021; Woo & Chan, 2020). Nevertheless, most of these studies focused on customers’ viewpoints to optimize customer relationship management and lacked investigation of employees’ perspectives. This represents a knowledge gap and an important area for further investigation, given the inevitability of emotional contagion in the service sector and the magnitude of its effects on employees’ behavior and well-being, work outcomes, and the hotel’s overall performance. Moreover, prior studies focused mainly on the consequences of emotional contagion (Barsade et al., 2018; Kane et al., 2023; Zhang et al., 2023), rather than on developing effective strategies to control this phenomenon and manage its ramifications. As best as the authors can ascertain, very limited research has investigated LMX as an alleviator that reduces the negative outcomes of emotional contagion on employees’ performance and well-being in hotel settings, underscoring another under-researched aspect of emotional contagion.

This study draws on JD–R and COR theories to propose a model examining (1) the direct effects of emotional contagion on psychological well-being, job performance, and turnover intentions; (2) the mediating role of well-being; and (3) the moderating role of LMX on associations between emotional contagion and employees’ psychological well-being, job performance, and intentions to leave. In doing so, it advances hospitality management research by clarifying how emotional contagion shapes employee outcomes in emotionally demanding contexts and by underscoring LMX’s role in mitigating its negative effects.

## 2. Literature Review

### 2.1. Emotional Contagion

Emotion is defined as an adaptive response to environmental demands that focuses our attention on the most pressing issues and prepares us to act (Elfenbein, 2023). According to component-process theories of emotion, people experience specific emotions in response to noticing and evaluating inputs for their subjective meaning (Scherer & Moors, 2019). The concept of emotional contagion was first introduced by McDougall in 1923, but the term was later interpreted by (Aldao et al., 2015) to describe the process by which individuals “catch” the emotions of those around them. Emotional contagion refers to the tendency to unconsciously imitate and synchronize with the body language and expressions of others, including facial gestures, speaking tones, body postures, and movements, leading to the convergence of emotions (Petitta et al., 2021). Emotional contagion occurs when an individual’s emotions spread to others. People with high emotional contagion feel the same emotions as others and react sentimentally to their situations (Petitta et al., 2021). Multiple processes facilitate emotion contagion. People can unintentionally imitate others’ emotional behaviors and expressions through unconscious emotional synchronization, leading to shared emotional experiences. Empathy also facilitates emotional exchange by enabling people to cognitively mimic others’ emotional states and experience similar emotions (Barsade & Gibson, 2007).

Furthermore, individuals accelerate their awareness and perception of others’ emotions through verbal communication, body language, and facial expressions. For instance, if one team member exhibits fear, the other members will also experience the same emotion. Team members who connect with the group and its objectives experience strong emotional contagion (Kane et al., 2023). For teams to achieve common goals, members require shared emotional experiences that reinforce collective values and behaviors (Janelle et al., 2020). Such emotions shape interpersonal relations, coordination, problem-solving, and cohesion (van Kleef & Fischer, 2016). In service organizations, emotional contagion strongly affects employee well-being and outcomes, as workers continuously adapt their emotions during interactions with colleagues, superiors, and customers. Negative emotions, however, often spread and lead to burnout, emotional exhaustion, and reduced commitment (Barsade et al., 2018).

### 2.2. Psychological Well-Being

Well-being is characterized by a person’s positive cognitive–evaluative attitude toward oneself and the outside world, as well as a sense of personal fulfilment derived from his/her actions (Cooke et al., 2016). There are two types of psychological well-being: hedonic and eudemonic (Guest, 2017). The hedonic aspect of psychological well-being focuses on happiness and is associated with job satisfaction, pleasant emotions at work, and the harmony of optimistic and pessimistic thought patterns (Edgar et al., 2021). The fulfilment of one’s potential and successful functioning at work are considered key aspects of an employee’s eudemonic well-being. It involves sentiments of involvement and affective commitment to the company and is linked to discovering meaning and purpose in one’s work (Marescaux et al., 2019). According to (Kundi et al., 2021), business organizations can benefit from fostering employees’ psychological well-being, as it has a favorable influence on work-related attitudes and behaviors.

### 2.3. Job Performance

Job performance is employees’ measurable activities, behaviors, and outputs that directly contribute to achieving organizational goals and success (Shin & Konrad, 2017). It is the physical behavior of employees in their pursuit of organizational goals. In other words, job performance encompasses all actions employees undertake as part of their responsibilities and duties within the organization (Jex & Britt, 2014). Prior research has emphasized the importance of employee performance, which is directly linked to organizations’ overall performance and productivity (Gencer et al., 2023; Shin & Konrad, 2017). This has encouraged many scholars to explore the drivers of employee performance. Job conditions and organizational characteristics are recognized as essential predictors of job performance (Diamantidis & Chatzoglou, 2019).

### 2.4. Intentions to Leave

Intention to leave has been recognized as a precursor to turnover (Kim et al., 2010). Thus, an employee’s intention to leave the job has a significant impact on the actual quitting process within organizations. Employee turnover is costly for organizations and involves two categories of expenses: direct and indirect. The direct costs include the expenses of recruiting, selecting, and hiring new personnel to fill the positions left by departing employees. The indirect costs stem from the long-term consequences of turnover, which affect organizational performance, such as decreased morale and increased workload on current employees (Guzeller & Celiker, 2019). Additionally, the stress of increased workload, in the form of longer work hours and additional responsibilities, can lead to lower service quality and higher operating expenses due to the hiring of temporary employees (Le et al., 2023).

### 2.5. Leader–Member Exchange (LMX)

According to Yu et al. (2018), LMX quality reflects how well parties regard the relationship between a leader and a follower. It is the positive reciprocal rapport between supervisors and employees (Jung & Yoon, 2019). A fundamental premise of the LMX theory is that leaders cultivate unique connections of different caliber with their team members. This is called LMX differentiation, in which leaders establish distinct quality-exchange connections. This connection might be seen as high-quality when marked by mutual respect, trust, and consideration for followers’ needs. The LMX connection, on the other hand, may be seen as a low-quality partnership in which the emphasis is on the details listed in the official job description, with minimal support and involvement. According to Liang et al. (2022), a person’s relationship with their leader can be a significant source of well-being. Accordingly, followers’ individual work stress is decreased by excellent LMX quality. Perceived social support in the form of LMX is linked to satisfaction and well-being (Siedlecki et al., 2014). According to Gariépy et al. (2016), social support can promote well-being both directly and indirectly by strengthening social bonds and serving as a buffer against stress.

## 3. Hypotheses Development

### 3.1. Job Demand–Resource (JD–R) Theory and Emotional Contagion Outcomes

The JD–R model is a well-known framework in occupational stress research for understanding the elements that influence workers’ well-being and job performance. According to the JD–R model, each job has unique demands (e.g., workload, time constraints, and emotional needs) and resources (e.g., social support, autonomy, and development opportunities). According to this model, when high job expectations are not balanced with adequate resources, workers may experience stress, burnout, and poor health issues. When employees have enough job resources, their motivation, engagement, and overall work performance will improve. Accordingly, the JD–R model focuses on a broader range of needs and resources when predicting and explaining workers’ well-being and performance (Demerouti et al., 2001).

Emotional contagion echoes the staff’s tendency to mirror and absorb coworkers’ emotions. People high in emotional contagion repeatedly struggle to control their own feelings, making people more vulnerable to their peers’ emotional states (Aldao, 2013). Research showed that while emotional contagion is a key issue affecting affective operations, its influence on well-being depends on the efficiency of emotional regulation. Poor regulation under high contagion can trigger negative emotions such as anger or anxiety, which, if prolonged, may lead to strained interactions, health problems, and reduced resilience (Verzeletti et al., 2016). Thus, the following hypothesis can be assumed:

**H1.**  *Emotional contagion negatively relates to employees’ psychological well-being.*

Psychological and physical well-being are greatly influenced by the emotions experienced and expressed. In these situations, controlling one’s emotions is essential for responding to environmental demands (Janelle et al., 2020). For example, emotions can help employees achieve their goals, improve interpersonal relationships, and influence their behavior and performance. It is anticipated that unpleasant feelings arising from emotional contagion may lower job attitude appraisal and potentially deplete the resources needed to perform better (Reizer et al., 2019). Studies on the outcomes of emotional contagion in the service industry revealed that when people interact with others in the same network, their thoughts, attitudes, moods, and emotions are influenced (Barsade et al., 2018). For this reason, it has been determined that social and organizational factors play a crucial role in shaping people’s attitudes toward their jobs and their behavior, even to the point of turnover. Given that emotional contagion involves imitating the positive or negative emotions of others, it can affect human behavior in the workplace (Ryan et al., 2021). Therefore, this study posits the subsequent hypothesis:

**H2.**  *Emotional contagion negatively relates to job performance.*

Employees who are subjected to high job demands, including emotional demands, are more likely to experience job burnout and quit their jobs (Chênevert et al., 2021). It is assumed that emotional contagion is a human flaw and that people who are easily excited or agitated are less capable of coping with demanding situations (Petitta et al., 2021). According to the JD–R model, a desire to disengage from work arises when insufficient resources are available to fulfil job needs. When faced with heavy demands, employees may consider quitting or seeking a role change. In addition, excessive workloads, including emotional demands and contagion, can cause job stress and employee alienation (Dodanwala et al., 2023). Emotional demands and contagion can lead to negative feelings, prompting people to turn to behavioural coping mechanisms, such as evading reality or withdrawing from stressful situations to manage their emotions (Folkman, 2020). Therefore, it can be hypothesized that:

**H3.**  *Emotional contagion positively relates to the employee’s intention to leave.*

### 3.2. Psychological Well-Being, Job Performance, and Intentions to Leave

Well-being can be viewed through two dimensions: hedonic well-being, which reflects life satisfaction, and eudaimonic well-being, which relates to functioning and realizing one’s potential (Keyes, 2006). Self-determination theory emphasizes that autonomy, competence, and relatedness are core drivers of eudaimonic well-being, fostering intrinsic motivation and better performance. When these demands are met, workers have more energy, higher levels of intrinsic motivation, and higher levels of cognitive engagement, all of which improve job performance. Simultaneously, the hedonic dimension (life pleasure and good affect) enhances mood, lowers stress, and expands workers’ thought–action repertoires, allowing for more adaptable problem-solving and thinking (Cerasoli et al., 2016; Ryan & Deci, 2000). In parallel, research shows that employee well-being enhances performance and productivity (Hewett et al., 2018), engagement (Tisu et al., 2020), customer satisfaction (Sharma et al., 2016), and organizational citizenship behavior (Mousa et al., 2020). Moreover, well-being is strongly linked to individual performance and organizational survival (Wright & Huang, 2012). Therefore, it can be posited that psychological well-being positively influences employees’ performance.

**H4.**  *Psychological well-being affects job performance positively.*

According to Xue et al. (2022), employee well-being is a key aspect of a positive work environment. Their study concluded that organizational members’ psychological well-being was essential for reducing employees’ tendency to leave. Studies indicate that employees’ well-being is important to business organizations, as it affects many work-related outcomes, such as absenteeism, turnover, and health-related expenses (Kuvaas & Dysvik, 2010), as well as work performance (Wright & Huang, 2012). Other studies show that employees’ decisions to leave or remain in their current positions are influenced by their well-being (Sivapragasam & Raya, 2018). Hence, it can be assumed that:

**H5.**  *Psychological well-being affects intention to leave negatively.*

The above literature suggests that emotional contagion influences employees’ psychological well-being, which in turn affects other outcomes, including job performance and intentions to leave the job. In other words, the psychological well-being of frontline employees can mediate the linkages between emotional contagion and both employees’ performance and intentions to leave. Prior research showed that workers who maintained better well-being conditions have performed well at work (Kalliath et al., 2017). Likewise, (Liang et al., 2022) found that high levels of well-being were associated with a significant improvement in organizational and job performance. Jones et al. (2015) reported that some aspects of psychological well-being, such as greater self-awareness, goal setting, autonomy, and use of strengths, may have a favorable effect on job satisfaction. Accordingly, the subsequent hypotheses are postulated:

**H6.**  *Employees’ psychological well-being mediates the linkage between emotional contagion and job performance.*

**H7.**  *Employees’ psychological well-being mediates the linkage between emotional contagion and intentions to leave.*

### 3.3. Conservation of Resources Theory (COR) and the Moderating Effects of LMX

From the JD–R perspective, emotional contagion is an emotional demand that can hinder performance and increase turnover intentions unless balanced by resources. Leader–member exchange (LMX) serves as such a resource, buffering the strain of contagion. To clarify this moderating role, conservation of resources (COR) theory is applied, which posits that individuals seek to acquire, protect, and invest resources—such as supportive ties with supervisors—that help sustain well-being and performance (Halbesleben et al., 2014; S. E. Hobfoll et al., 2018). The COR theory also offers a helpful framework for analyzing the relationship between resource availability or scarcity and employee well-being (S. E. Hobfoll, 1989; S. E. Hobfoll et al., 2018). In line with COR theory, employees use available resources, such as LMX, to obtain greater value and protect themselves from harmful stress that could jeopardize those values, i.e., well-being in this case (S. Hobfoll, 2014). Thus, service organizations need to establish resource passageways for workers to preserve and improve their well-being, particularly through the actions of their managers and supervisors (S. E. Hobfoll, 2011). This paper views powerful LMX as a contextual internal resource that offers psychological well-being. Grounded in the COR theory, staff are likely to reinvest such resources to protect well-being, allocating LMX as a main mechanism for sustaining it.

Effective interrelationships with superiors and peers can improve staff well-being by promoting reciprocity and trust (Cropanzano et al., 2017; Nisar & Maroulis, 2017). High-quality LMX interrelationships, built on mutual trust and positive interactions, serve as a key organizational internal resource for supporting employees (Dulebohn et al., 2012). Prior studies show that supervisor and organizational support positively influence psychological well-being (Mousa et al., 2020), with social support linked to greater mindfulness, self-compassion, and positive well-being, as well as reduced stress and depression (Wilson et al., 2020). Based on these findings, social support through LMX may serve as a moderator, strengthening the link between workplace experience and employee well-being. Subsequently, the following moderation effect can be assumed:

**H8a.**  *LMX moderates the linkage between emotional contagion and psychological well-being.*

**Figure 1:**
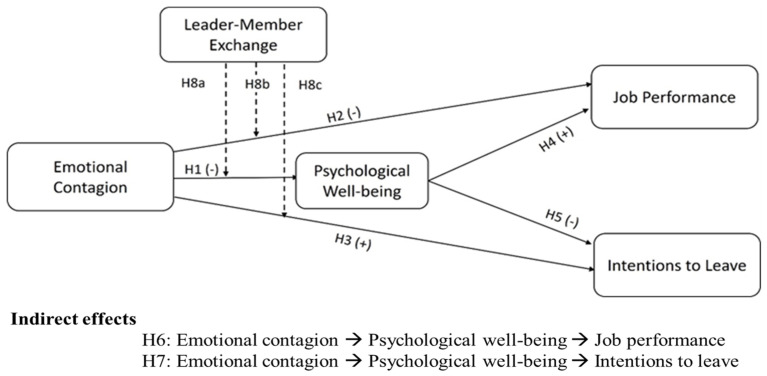
Conceptual model.

LMX can also help enhance employees’ performance and reduce their intentions to leave the job. In other words, a high-quality relationship between the leader and team members is characterized by support, trust and mutual respect. Such qualities make employees feel valued and supported by the organization, which, in turn, boosts their performance and commitment. On the other hand, poor leader–member rapport results in lower performance and higher turnover. Mello and Tomei (2021) reported that organizational support aimed at improving workers’ work–life balance led to better employee performance. According to Maertz et al. (2007), employees’ leave intentions were significantly intervened by perceived positive supervisory support. Petitta and Naughton (2015) also concluded that perceived managerial support shaped employees’ work attitudes, which in turn affected their commitment and intentions to leave. Hence, this study posits the last hypotheses (Figure 1 illustrates all the study hypotheses):

**H8b.**  *LMX moderates the linkage between emotional contagion and job performance.*

**H8c.**  *LMX moderates the linkage between emotional contagion and intention to leave.*

## 4. Research Methods

### 4.1. Instrument and Measures

The measurement items employed in this study were drawn from well-established scales in prior research. Emotional contagion was assessed using six items from Mehrabian and Epstein’s scale (Mehrabian & Epstein, 1972), which had been previously adapted by Petitta and Naughton (2015) and later applied in the hospitality context by Elshaer et al. (2025) and Aboutaleb et al. (2025). Employees’ psychological well-being (PW) was measured with a 10-item instrument developed by Pradhan and Hati (2022). Job performance (JP) was measured using a 5-item scale initially introduced by Reizer et al. (2019), a measure widely used in hospitality research (Karatepe et al., 2006; Verma et al., 2024). Turnover intention, or intention to leave (IL), was measured using three items from Scherer and Moors (2019), a scale recognized as one of the most commonly used measures in this domain (Nguyen et al., 2023). Leader–member exchange (LMX) was assessed using five items developed by Borchgrevink and Boster (1997), which have also been adopted in hospitality studies, including Jung and Yoon (2019).

All constructs, except demographic variables, were measured on a 5-point Likert scale ranging from 1 (strongly disagree) to 5 (strongly agree). To ensure content validity, the survey instrument was reviewed by 11 academic experts and 12 industry professionals. Importantly, the integrity and essence of the original scales were maintained throughout the adaptation process.

### 4.2. Participants and Procedures

Data were collected using online surveys (Microsoft Forms v365) distributed via hotel managers/HR in Sharm El-Sheikh’s high-rated hotels. From May to August 2024, 823 questionnaires were returned, of which 792 were valid. Participants were informed that their involvement was voluntary and that they had the right to withdraw at any time during the study. They were also assured that the collected data would be analyzed statistically for research purposes only and managed in complete privacy. Furthermore, respondents were informed that by completing the survey, they were offering their informed consent. The sample involved 72.2% of males and 27.8% of females, age levels were from 18 to 60+, with balanced educational levels (49.9% middle school level, 34.1% bachelor’s level) and marital status (56.6% were married, 41.4% were single). Almost 40% of the participants had over 10 years of experience (Table 1).

### 4.3. Data Analysis

The justified hypotheses were evaluated using PLS-SEM with SmartPLS 4.0, and descriptive statistics were conducted with SPSS 24.0. The conduct of PLS-SEM was appropriate, as the main objective of this paper was to predict relationships among latent variables. This stands in contrast to CB-SEM, which is typically employed to confirm established theoretical relationships. Likewise, unlike CB-SEM, PLS-SEM is more efficient and stable for complex models with many diverse latent variables. It also requires fewer data assumptions and flexibly handles various sample sizes. PLS-SEM is also a non-parametric approach, which is appropriate for data that do not adhere to a multivariate normal distribution, in contrast to CB-SEM (Hair et al., 2019). The analysis followed the standard two-stage procedure recommended by Hair et al. (2017), which involves evaluating both the measurement (outer) model and the structural (inner) model.

## 5. Results

### 5.1. Test of Common Method Bias (CMB) and Normality

According to Harman’s single-factor analysis, when the total variance of a single factor exceeds 50%, CMB exists. The results revealed that one factor can explain about 25.456% of the total variance. Hence, CMB was not a concern (Podsakoff et al., 2003). The study also tested the “variance inflation factor” (VIF) values to calculate CMB. All VIF values were below the recommended threshold of 3.3, ranging from 1.157 to 1.418 (Kock, 2015). This indicated that there were no significant collinearity concerns. In addition, the data’s normality was examined using skewness and kurtosis statistics. The absolute values for all items were below the recommended cutoffs of 2 for skewness and 7 for kurtosis (Curran et al., 1996), thereby confirming that non-normality did not pose an issue (see Table 2).

### 5.2. Reliability and Construct Validity

According to Hair et al. (2019), several criteria are recommended for assessing the “convergent validity” (CV) of the measurement model in PLS-SEM, including indicator loadings (λ), Cronbach’s alpha (α), and “composite reliability” (CR). The accepted thresholds for these indices are ≥0.70, and the “average variance extracted” (AVE) should be >0.50. As shown in Table 2, the measurement model meets all requirements, thereby confirming adequate convergent validity and demonstrating the internal consistency of the items for each construct.

With respect to discriminant validity (DV), Fornell and Larcker (1981) proposed that the AVE of each construct should be greater than the squared interconstruct correlations. The results in Table 3 indicate that this condition is met, thus providing evidence of discriminant validity. In addition, the “heterotrait–monotrait ratio” (HTMT) was examined, as suggested in more recent research. The findings presented in Table 4 demonstrate that all HTMT values are below the 0.90 threshold (Gold et al., 2001), further confirming discriminant validity.

### 5.3. Structural Model and Testing Hypotheses

To evaluate the structural model, several criteria were examined, including variance inflation factors (VIFs), coefficient of determination (R^2^), predictive relevance (Q^2^), and path coefficients (β) (Hair et al., 2019). To rule out multicollinearity among constructs, inner VIFs should be below 3.0, while outer item VIFs should remain below 5.0. In addition, R^2^ values must align with established academic standards and the study’s contextual requirements. Path coefficients (β) should be statistically significant, and Q^2^ values should exceed 0.0 to confirm predictive relevance (Hair et al., 2019).

As presented in Table 5, all inner VIF values were below 3.0, while outer VIFs ranged between 1.614 and 3.857, remaining under the recommended threshold. These results confirm that multicollinearity was not a concern in the model. In terms of explanatory power, the R^2^ value for psychological well-being was 0.295, indicating that 29.5% of its variance was explained by the predictor constructs within the structural model. Similarly, job performance recorded an R^2^ of 0.538, while intention to leave showed an R^2^ of 0.235; both exceed the minimum acceptable benchmark of 0.10. Additionally, Q^2^ values were greater than 0.0, thereby supporting the model’s predictive relevance. Path coefficients (β) were also found to be statistically significant at the 0.01 level. Finally, the overall goodness-of-fit (GoF) of the PLS-SEM model can be assessed using the formula proposed by Tenenhaus et al. (2005):

$$GoF=\sqrt{{AVE}_{avy}\times {R^{2}}_{avy}}$$

The GoF is low, medium, and high at 0.1, 0.25, and 0.36, respectively. GoF for our model is high, at 0.499.

Based on the evidence for the measurement outer and structural inner model benchmarks’ validity, we tested our hypotheses (Table 5).

**Figure 2:**
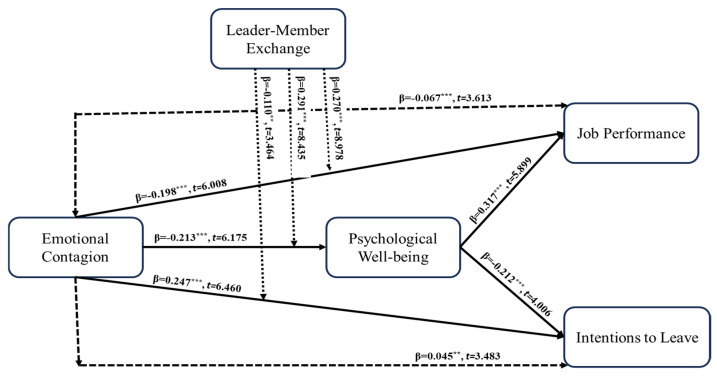
Estimation of structure model. Note: ** significant level less than 0.05; *** significant level less than 0.005.

As illustrated in Table 5 and Figure 2, emotional contagion (EC) was found to influence psychological well-being (PW) significantly (β = −0.213; t = 7.677; *p* < 0.001), job performance (JP) (β = −0.198; t = 6.008; *p* < 0.001), and intention to leave (IL) (β = 0.247; t = 6.460; *p* < 0.001), thereby supporting H1, H2, and H3. Furthermore, PW exerted a positive effect on JP (β = 0.317; t = 5.899; *p* < 0.001) and a negative effect on IL (β = −0.212; t = 4.006; *p* < 0.001), confirming H4 and H5. In addition, mediation analysis revealed that PW partially mediated the relationships between EC and JP (β = −0.067; t = 3.613; *p* < 0.001) and between EC and IL (β = 0.045; t = 3.483; *p* < 0.001), thereby providing support for H6 and H7.

**Figure 3:**
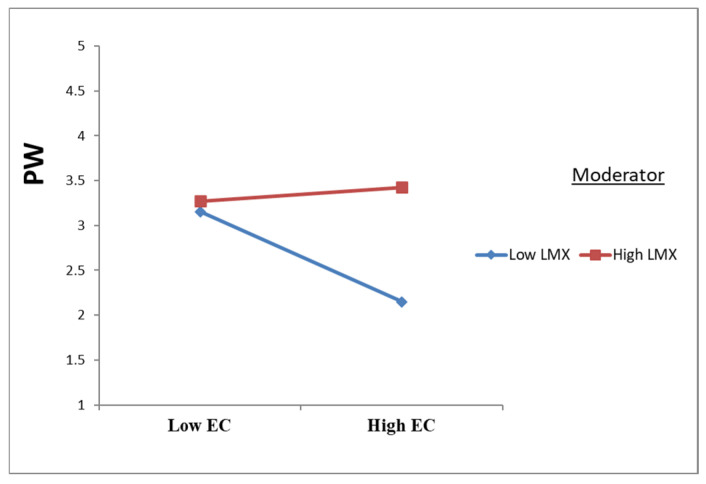
Moderation effects of LMX on EC to PW.

**Figure 4:**
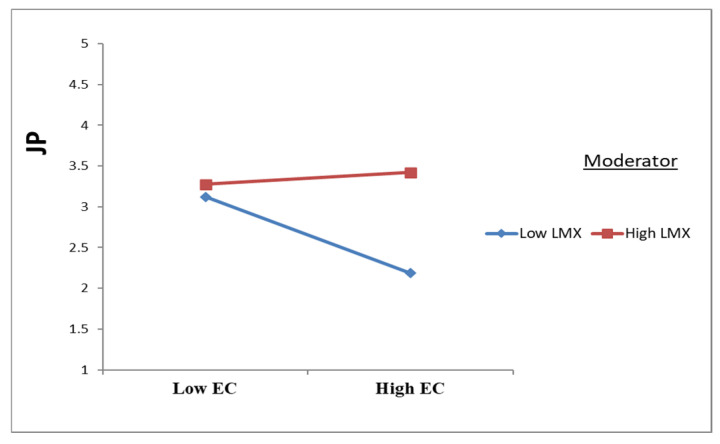
Moderation effects of LMX on EC to JP.

**Figure 5:**
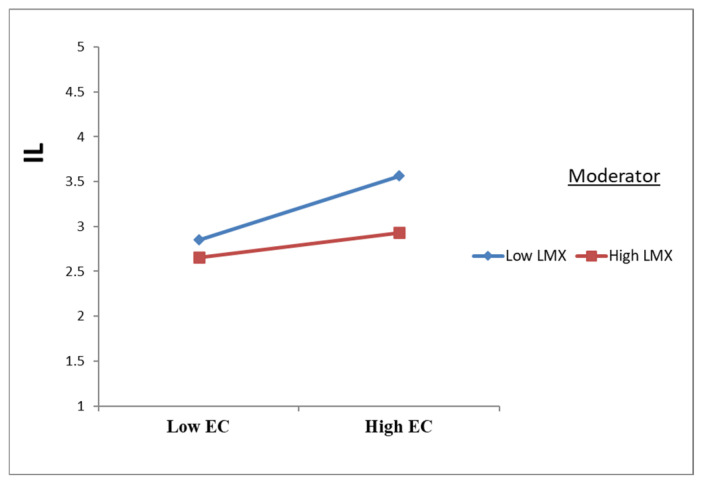
The moderating effects of LMX on EC to IL.

As presented in Table 5 and Figure 3, Figure 4 and Figure 5, leader–member exchange (LMX) significantly moderated the relationships between emotional contagion (EC) and the study outcomes. Specifically, LMX attenuated the negative effect of EC on psychological well-being (PW) (β = 0.291; t = 8.435; *p* < 0.001) and on job performance (JP) (β = 0.270; t = 8.978; *p* < 0.001), while also weakening the positive effect of EC on intention to leave (IL) (β = −0.110; t = 3.464; *p* = 0.001). These findings provide empirical support for H8a, H8b, and H8c.

## 6. Discussion

### 6.1. Findings and Contribution to Theory

This study has three aims: (1) to test the direct impacts of emotional contagion on psychological well-being, job performance, and intention to leave, moderated by LMX; (2) to test the indirect influence of emotional contagion on performance and intention to leave through psychological well-being; and (3) to assess how psychological well-being influences both performance and turnover intention. This paper reveals that emotional contagion negatively affects psychological well-being, which agrees with Xerri et al. (2023), and job performance, concurring with the study of Ryan et al. (2021). However, emotional contagion positively affects the intention to leave, consistent with Feng and Cui (2024). Psychological well-being mediates the relationships between emotional contagion and both job performance, which concurs with Kalliath et al. (2017), and intention to leave, which coincides with Sivapragasam and Raya (2018).

JD–R theory was adopted to illustrate these results. This theory postulates that every occupation has demands that negatively affect employees’ attitudes and behaviors as they try to cope with them. In this theory, emotional contagion can be viewed as a source of stress, as employees with high emotional contagion are unable to control their feelings when exposed to others’ emotions. That is, employees with high emotional contagion cannot control their emotions (Aldao et al., 2015). This directs employees to exhibit negative attitudes and behaviors, resulting in low performance, low psychological well-being, and high turnover. This study expands on JD–R theory by explicitly connecting emotional experiences to job results and well-being, by defining emotional contagion as an affective job demand that may deplete employees’ psychological resources. This study emphasizes the importance of interpersonal emotional dynamics in influencing employee functioning, in contrast to previous hospitality studies that primarily focus on workload or emotional labor (Grobelna, 2021; Vashdi et al., 2022).

The study also reveals that LMX could moderate the effects of emotional contagion on psychological well-being, job performance, and intentions to leave. The moderating role of LMX between emotional contagion and psychological well-being is consistent with the study by Fatima et al. (2018). LMX’s moderating role between emotional contagion and job performance was confirmed by Mello and Tomei (2021). Additionally, the moderating role of LMX between emotional contagion and the intention to leave is consistent with Kuvaas and Dysvik (2010). These findings underscore the critical role of leaders’ interpersonal skills, including emotional intelligence, empathy, and supportive communication. Robust and high-quality rapport between leaders and team members acts as a protective resource that buffers consequences of negative emotions transfer in the workplace. That is, strong LMX can facilitate the process of emotional regulation and improve the cognitive appraisal of emotions, which minimizes the transmission of negative emotions or lessens their effects on employees’ well-being.

COR can be used to elaborate on these results. JD–R assumes that every occupation has its demands, including emotional demands stemming from emotional contagion, which can cause negative behaviors and attitudes. Therefore, job demands can be buffered using adequate resources. COR represents a motivational theory that explains how people allocate and conserve resources (S. E. Hobfoll et al., 2018). According to COR, people are motivated to acquire and protect job-related resources, such as connections with superiors (Halbesleben et al., 2014). LMX serves as a contextual resource that fosters psychological well-being and reduces turnover intentions. Aligned with COR theory, high-quality LMX—built on support, trust, and respect—encourages employees to reinvest resources, enhancing performance and commitment, whereas poor LMX leads to low performance and higher turnover. Accordingly, this study contributes to the literature by positioning high-quality leadership and LMX as an essential aspect of maintaining employees’ well-being and improving their job performance through preventing emotional contagion from becoming a strain or additional job demand. The study offers a resource-based explanation for these effects by utilizing COR theory, demonstrating how psychological well-being serves as a crucial resource moderating the influence of emotional contagion on work performance and turnover intentions. By connecting affective mechanisms with resource-based processes, this integration goes beyond previous studies (Kruesi & Bazelmans, 2023) and clarifies how employee behaviors in emotionally demanding situations are influenced by cycles of resource gain and loss (Elshaer et al., 2024).

This study also offers a more thorough theoretical explanation of how emotional demands might result in diverse behavioral consequences by looking at parallel but different outcomes (performance vs. intention to leave). By combining emotional, resource-based, and relational approaches, these contributions together enrich hospitality management research and provide a conceptually deeper and more predictive model than previous studies.

In conclusion, although the results support the mediation and moderation effect, we should interpret these relationships within the context of the study’s cross-sectional design. As highlighted by Maxwell and Cole (2007) and Aguinis et al. (2017), measuring variables at a single point in time limits the ability to definitively establish temporal precedence and causal inference. Nonetheless, our findings can serve as a basis for future longitudinal validation because they are grounded in a robust theoretical background and provide a significant snapshot of the current dynamics in the Egyptian hotel industry.

### 6.2. Practical Implications

This paper offers practical suggestions for managing emotional contagion among hotel frontline staff. As contagion is predictable, hotels should promote a positive workforce environment that fosters optimism and enthusiasm, thus improving well-being and job performance. This can be achieved by ensuring sufficient internal resources, supporting collaboration, addressing concerns promptly, encouraging work–life balance, and cultivating a supportive organizational culture.

Moreover, sustaining staff psychological well-being should be a high priority for HRM in hotels, given its impact on job performance and turnover ratios. This can be conducted by safeguarding access to mental health resources and promoting psychological support systems, such as internal counselling services, wellness programs, and stress management initiatives. Furthermore, hotel top managers can take proactive measures to prevent the escalation of emotional contagion among staff members. To that end, closely controlling emotional exchanges in the workplace and conducting standard well-being evaluations can enable supervisors or first-line managers to promptly address adverse emotions and promote the spread of positive opinions.

Similarly, staff development and training programs may help regulate emotional contagion in the working environment, including between managers and team members. Leadership training sessions can focus on developing leaders’ competencies to manage their own emotions, understand others’ emotions, respond appropriately to peers’ emotions, and navigate the spread of emotions within the team. Training sessions may also help frontline staff regulate their emotions, overcome negative emotions, and express positive feelings. Doing so enables hotels to cultivate a work environment saturated with positive emotions and to reduce the spread of pessimism among team members. More prominently, development sessions should also focus on fostering supportive networks among coworkers to help frontline staff share positive emotional practices, exchange emotional support, extend sympathy or empathy, uplift team morale, and sustain collective well-being.

Additionally, as this paper has confirmed, LMX can play a major role in mitigating the adverse impacts of emotional contagion. Hotel top managers should promote strong, reciprocal supervisor–employee relationships to manage emotionally demanding circumstances better. This can be achieved by offering psychological support, promoting mutual trust, encouraging communication and dialogue, establishing recognition and appreciation, granting resources and guidance, and supporting personal communication. Such methods help relieve stress, minimize emotional exhaustion, and avoid adverse psychological outcomes of emotional contagion.

Finally, promoting personalized LMX through a flexible leadership approach and customized organizational support may be highly helpful in managing emotional contagion. This entails managers tailoring their leadership style to their employees’ emotional needs and traits. It also demands regularly obtaining employees’ feedback on emotional situation, LMX, and organizational confirmation. This can shed light on leadership procedures that need enhancement and reflect the hotel’s commitment to staff well-being and retention.

### 6.3. Study Limitations and Directions for Future Research

This study has certain limitations. First, the results and implications of the study were based on hotel settings in Egypt, thereby the findings can be carefully generalized to similar cultures. Hence, future research in this area can integrate participants from multiple countries and examine the effects of cultural differences on the findings. Also, this study examined the role of one moderator, i.e., LMX, as an alleviator of emotional-contagion-related negative outcomes. Further studies can examine other or multiple moderators such as employees’ psychological resilience, emotional intelligence, or organizational support. Moreover, due to time and accessibility constraints, this study adopted a quantitative approach using a cross-sectional design. Future studies can yield a profound understanding of the dynamics of emotional contagion and possible ways to avoid its consequences by adopting a qualitative and/or longitudinal approach. Additionally, the gender imbalance in the sample (72% male) is a limitation. Given that emotional contagion and psychological well-being may be experienced differently across genders, the generalizability of the findings should be treated with caution. Thus, we suggest conducting comparative studies based on demographic data in the future.

## 7. Conclusions

Hotel frontline employees are susceptible to catching various emotions from co-workers and guests due to ongoing and direct interactions with them. Prolonged exposure to emotional contagion, particularly when negative emotions are reciprocated, can harm employees’ psychological well-being, negatively impact their performance, and drive them to leave the job. Encouragingly, the results support the hypothesis that the strong professional rapport and support inherent in LMX can help employees alleviate these negative outcomes and safely navigate this common phenomenon in service industry encounters.

## Figures and Tables

**Table 1:** Respondents profile.

Category	N = 792	Occurrence	%
Male/Female			
	[Male]	572	72.2
	[Female]	220	27.8
Age Level			
	[from 18 to 29]	347	43.8
	[from 30 to 39]	231	29.2
	[from 40 to 49]	171	21.6
	[from 50 to 59]	41	5.2
	From 60 and more	2	0.3
Education level			
	[High school level]	40	5.1
	[Middle school level]	395	49.9
	[Bachelor’s degree level]	270	34.1
	[Postgraduate degree]	30	3.8
	[Other]	57	7.2
Marital condition			
	Married	448	56.6
	Single	328	41.4
	Divorced	16	2.0
Experience years			
	Less than 2 years	161	20.3
	2 to 5	177	22.3
	5 to 10	140	17.7
	>10	314	39.6

**Table 2:** Confirmatory factor analysis results.

Factors and Items	λ	Mean	SD	SK	KU
EC: (α = 0.854, CR = 0.887, AVE = 0.566)					
EC1	0.801	3.182	1.335	−0.351	−1.109
EC2	0.805	2.390	1.127	0.562	−0.553
EC3	0.701	3.311	1.342	−0.561	−0.933
EC4	0.702	3.308	1.336	−0.614	−0.878
EC5	0.737	2.438	1.188	0.512	−0.711
EC6	0.762	3.061	1.251	−0.224	−0.949
PW: (α = 0.929, CR = 0.940, AVE = 0.610)					
PW1	0.770	4.221	0.756	−1.112	2.348
PW2	0.777	4.230	0.754	−1.222	2.871
PW3	0.781	4.187	0.816	−1.280	2.548
PW4	0.803	4.263	0.769	−1.291	2.924
PW5	0.824	4.274	0.740	−1.236	3.022
PW6	0.775	4.240	0.765	−1.236	2.832
PW7	0.786	4.244	0.774	−1.254	2.753
PW8	0.786	4.441	0.692	−1.413	3.139
PW9	0.701	4.521	0.723	−1.805	4.081
PW10	0.800	4.513	0.684	−1.687	4.100
JP: (α = 0.910, CR = 0.933, AVE = 0.735)					
JP1	0.877	4.177	0.918	−1.525	2.898
JP2	0.881	4.047	0.971	−1.141	1.298
JP3	0.794	3.904	1.058	−0.869	0.280
JP4	0.879	4.304	0.899	−1.839	4.124
JP5	0.851	4.251	0.916	−1.727	3.598
IL: (α = 0.901, CR = 0.938, AVE = 0.835)					
IL1	0.907	2.256	1.156	0.838	−0.031
IL2	0.927	2.246	1.070	0.809	0.243
IL3	0.908	2.355	1.144	0.717	−0.191
LMX: (α = 0.909, CR = 0.938, AVE = 0.754)					
LMX1	0.900	3.968	1.183	−1.387	1.160
LMX2	0.907	3.794	1.161	−1.067	0.456
LMX3	0.876	3.415	1.140	−0.571	−0.336
LMX4	0.904	3.725	1.154	−1.001	0.338
LMX5	0.741	3.242	1.203	−0.344	−0.842

Note: SK = skewness, KU = kurtosis; SD = standard deviation; CR = composite reliability; AVE = average variance extracted.

**Table 3:** Fornell–Larcker criterion.

Constructs	1	2	3	4	5
Emotional contagion	0.753				
2. Intentions to leave	0.331	0.914			
3. Job performance	−0.381	−0.356	0.857		
4. Leader–member exchange	0.248	−0.116	0.199	0.868	
5. Psychological well-being	−0.310	−0.385	0.594	0.140	0.781

**Table 4:** HTMT matrix.

Constructs	1	2	3	4	5
Emotional contagion					
2. Intentions to leave	0.342				
3. Job performance	0.398	0.394			
4. Leader–member exchange	0.326	0.122	0.206		
5. Psychological well-being	0.313	0.418	0.639	0.147	

**Table 5:** Hypotheses testing.

Hypothesis			[Β]	[T]	[P]	Results
Direct effect						
EC → PW			−0.213	6.175	***	H1-Supported
EC → JP			−0.198	6.008	***	H2-Supported
EC → IL			0.247	6.460	***	H3-Supported
PW →JP			0.317	5.899	***	H4-Supported
PW → IL			−0.212	4.006	***	H5-Supported
Indirect mediating effects						
EC → PW → JP			−0.067	3.613	***	H6-Supported
EC → PW → IL			0.045	3.483	**	H7-Supported
Moderating effects						
EC × LMX → PW			0.291	8.435	***	H8a-Supported
EC × LMX → JP			0.270	8.978	***	H8b-Supported
EC × LMX → IL			−0.110	3.464	**	H8c-Supported
IL	R2	0.235	Q2	0.182	Inner VIF	1.290
JP	R2	0.538	Q2	0.367	Inner VIF	1.291
PW	R2	0.295	Q2	0.163	Inner VIF	1.226

Note: Emotional contagion = EC; psychological well-being = PW; job performance = JP; intentions to leave = IL; leader–member exchange = LMX; *** significant level is <0.001; ** significant level is <0.01.

## Data Availability

The original contributions presented in the study are included in the article; further inquiries can be directed to the corresponding author.
